# Foreword

**DOI:** 10.1038/sj.bjc.6604570

**Published:** 2008-09-23

**Authors:** M A Richards

**Affiliations:** 1 Department of Palliative Medicine, 1st Floor, South Wing, St Thomas' Hospital, Lambeth Palace Road, London SE1 7EH, UK

This supplement of the *British Journal of Cancer* provides important new information and commentary on survival rates for the 20 most common cancers diagnosed in England and Wales over a 14-year period from 1986 to 1999. The analyses are based on data collected by cancer registries on 2.2 million cancer patients. The authors have assessed both trends in survival rates over time and inequalities in survival among groups of patients with different levels of deprivation, based on the postcode of residence for individual patients.

A particular feature of this publication is that for each cancer site the results and comments provided by epidemiologists are accompanied by a commentary from one or more leading clinicians. These clinical commentaries give insights into changes in the diagnosis and treatment of the relevant cancers and possible reasons for the changes observed over this time period.

Several key findings stand out from these papers:

- For most cancers, survival up to 10 years after diagnosis improved significantly between patients diagnosed in the mid-1980s and those diagnosed in the late 1990s. Marked improvements were observed for three of the most common cancers (breast, colorectal and prostate), though the remarkable improvement in prostate cancer survival rates might be due largely to improved case-finding.
- Important exceptions to this pattern of improved survival rates were observed. There was almost no change in the survival rate for lung cancer, pancreatic cancer and cervical cancer or bladder cancer.
- Survival rates were generally higher in more affluent than in more deprived groups. However, exceptions to this pattern were observed in the most recent time period (1996–1999) for brain tumours in men and ovarian cancer in women.
- The deprivation gap widened for most cancers between the mid-1980s and the late 1990s. It seems that more affluent groups benefit sooner from improvements in diagnosis and/or survival. However, deprivation gaps may be largely avoidable after a time lag. Testicular cancer provides an interesting example of this. Five-year relative survival rates in the most affluent groups were near to reaching the ceiling of 100% by 1996–1999. Survival rates for the most deprived groups seem to have been catching up.
- For almost all cancer types, inequalities were observed within 1 year of diagnosis. This may provide an opportunity for earlier monitoring of changes over time.

Interesting differences of opinion between epidemiologists and clinical commentators emerge in relation to interpretation of the reasons for the observed changes for several cancer types. In general, clinicians were likely to attribute the deprivation gap mainly to comorbidity. This interpretation is challenged by the epidemiologists who put more emphasis on late diagnosis in deprived groups as a cause for poorer survival.

These differences of opinion highlight the need for high-quality information on staging and comorbidity to be collected by clinical teams and to be transmitted to cancer registries. This is now being given high priority in England through the Cancer Reform Strategy. Understanding of the underlying reasons for the deprivation gaps is urgently needed so that NHS resources can be targeted most effectively.

Finally, these reports highlight the importance of comprehensive cancer registration to monitor cancer outcomes and as a tool for investigating the mechanisms underlying changes over time. To achieve the maximum benefit, clinicians and epidemiologists need to work in close partnership. This will be a major feature of the newly established National Cancer Intelligence Network in the United Kingdom.

MA Richards

